# *In Vitro* Inhibitory Effects of *Viburnum opulus* Bark and Flower Extracts on Digestion of Potato Starch and Carbohydrate Hydrolases Activity

**DOI:** 10.3390/molecules27103118

**Published:** 2022-05-13

**Authors:** Dominika Kajszczak, Agnieszka Kowalska-Baron, Dorota Sosnowska, Anna Podsędek

**Affiliations:** 1 Institute of Molecular and Industrial Biotechnology, Faculty of Biotechnology and Food Sciences, Lodz University of Technology, Stefanowskiego 2/22, 90-537 Łódź, Poland; dorota.sosnowska@p.lodz.pl; 2 Institute of Natural Products and Cosmetics, Faculty of Biotechnology and Food Sciences, Lodz University of Technology, Stefanowskiego 2/22, 90-537 Łódź, Poland; agnieszka.kowalska-baron@p.lodz.pl

**Keywords:** starch digestion, α-amylase, α-glucosidase, bark, flower, *Viburnum opulus*, phenolic compounds

## Abstract

One of the effective treatments for diabetes is to reduce and delay the absorption of glucose by inhibition of α-amylase and α-glucosidase in the digestive tract. Currently, there is a great interest in natural inhibitors from various part of plants. In the present study, the phenolic compounds composition of *V. opulus* bark and flower, and their inhibitory effects on *in vitro* potato starch digestion as well as on α-amylase and α-glucosidase, have been studied. Bark and flower phenolic extracts reduced the amount of glucose released from potato starch during tree-stage simulated digestion, with IC_50_ value equal to 87.77 µg/mL and 148.87 µg/mL, respectively. Phenolic bark extract showed 34.9% and 38.4% more potent inhibitory activity against α-amylase and α-glucosidase, respectively, but the activity of plant extracts was lower than that of acarbose. Chlorogenic acid (27.26% of total phenolics) and (+)-catechin (30.48% of total phenolics) were the most prominent phenolics in the flower and bark extracts, respectively. Procyanidins may be responsible for the strongest *V. opulus* bark inhibitory activity against α-amylase, while (+)-catechin relative to α-glucosidase. This preliminary study provides the basis of further examination of the suitability of *V. opulus* bark compounds as components of nutraceuticals and functional foods with antidiabetic activity.

## 1. Introduction

The escalating tendency in the prevalence rate of diabetes complications hints that recent medical treatments for the management of diabetes are not adequate, and the use of additional treatments could raise the validity of diabetes management. Type 2 diabetes is the most common form of the diabetes (90–95% of all cases) and is characterized by elevated postprandial blood glucose levels [1,2]. One of the acceptable ways to reduce hyperglycemia is by retarding the actions of carbohydrate hydrolyzing enzymes and, consequently, reducing the carbohydrate digestion and absorption of glucose by the brush border [3]. Much effort has been extended in search of effective carbohydrate hydrolases inhibitors from the plants in order to develop functional food or to introduce a natural antidiabetic supplements, and to discover of novel therapeutic agents [2,4]. So far, structurally diverse groups of compounds derived from different morphological parts of plants were analyzed for the ability to inhibit the activity of α-amylase and α-glucosidase [3,5,6,7,8,9,10,11,12]. The above inhibitory activity was demonstrated by various phytochemicals, for example essential oils, organosulfur compounds, betaines, terpenoids, saponins, phytosterols, and alkaloids [7]. Nevertheless, most studies attribute this activity to phenolic compounds. These large group of secondary plant metabolites, based on *in vitro* studies, clinical trials, and some animal models, have been proposed as effective agents in the treatment of diabetes and as prevention of its long-term complications [13,14,15,16,17]. Moreover, the plant-derived inhibitors are more acceptable due to their low cost, and lower amount of side effects than the commercial inhibitors such as acarbose and voglibose, which have serious gastrointestinal side effects like diarrhea, flatulence, bloating, etc. [18].

Our *in vitro* cell-based studies demonstrated that phenolic rich fraction obtained from *V. opulus* fruit juice and extract from the remaining pomace decreased the uptake of fluorescent glucose analogue 2-(*N*-(7-nitrobenz-2-oxa-1,3-diazol-4-yl)amino)-2-deoxyglucose by human adenocarcinoma Caco-2 cells [19], and decreased glucose-stimulated insulin secretion in the mouse insulinoma cell line MIN6 [20]. Additionally, a screening study with cell-free assay identified *V. opulus* fruit pulp acetone extract as inhibitors of carbohydrates hydrolyzing enzymes (α-amylase and α-glucosidase) or protein tyrosine phosphatase, which is known as the major negative regulator in insulin signaling [21]. Our recent research has also shown that the ingredients of *V. opulus* dried fruit are inhibitors of α-amylase and α-glucosidase activity, and delay the formation of glycation end products [22]. *In vivo* study on *V. stellato-tomentosum* aerial parts has demonstrated that supplementation of the ethanol extract at 150 mg/kg dose one a day for 17 weeks significantly decreased fasting glucose insulin in a homeostasis model assessment of insulin resistance in high-fat diet fed C57BL/6J mice [23].

To the best of our knowledge, this type of research is lacking for other parts of the *V. opulus* such as the bark and flowers. In Poland, both plant materials are commercially available in the form of droughts. *V. opulus* shrub is common in natural habitats of Western and Central Europe, Asia, Caucasus, and Asia Minor [24]. Its white flowers are produced in corymbs 4–11 cm in diameter at the top of the stems. Each bloom is composed of an outer ring of large sterile flowers and an inner ring of tiny fertile ones. The decorative cultivar ‘Roseum’ (synonym ‘Sterile’, ‘Snowball’) has only sterile types of flowers that give the appearance of snowballs. The bark of *V. opulus* is green-brown on the outer surface and green-yellow to red-brown on the inner surface, and is harvested in spring and summer when the plant is flowering. It has a strong characteristic odor and tastes somewhat bitter [25]. The bark (Cortex Viburni) of the *V. opulus* species is used for medicinal purposes (2% infusion, decoction, or hydroalcoholic extracts) in the treatment of stomach or uterine bleeding and hemorrhoids [26]. There are several research studies about bioactive compounds and biological activity of fruit, but very few literature data concerning the *V. opulus* bark and flower [25,27]. So far, bark was characterized by a higher level of total phenolics and tannins as compared to fruit and flower, while flowers had the lowest [28,29]. The previous studies have shown the presence of hydroxycinnamic, benzoic and phenylacetic acids derivatives, and flavanols with favalignans in bark [30,31]. Moreover, in our earlier work, hydroxycinnamic acids, flavanols, and flavonols were identified in flowers [28]. 

The major aim of the present *in vitro* study was to investigate the effects of *V. opulus* bark and flower phenolic extracts on potato starch digestion, and on the activity of α-amylase and α-glucosidase. The hydrolytic activity of both enzymes was also tested in the presence of water and acetate fractions separated from the extracts. As the pharmacological activity of plant extracts is related to the presence of phenolic compounds, the phenolic profiles of extracts and fractions were also determined using ultrahigh-performance liquid chromatography-mass spectrometry (UPLC-MS).

## 2. Results and Discussion

### 2.1. Phenolic Profiles of the Bark and Flower Extracts and Fractions

**Figure 1:**
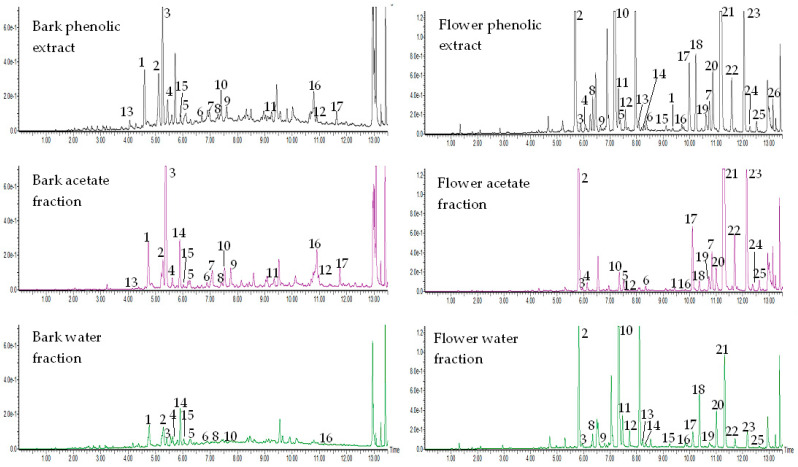
UPLC chromatograms of *V. opulus* bark and flower phenolic extracts and fractions. Refer to Table 1 and Table 2 for the identification of each numbered peak of bark and flower samples, respectively.

So far, most of the research have been carried out to characterize the phenolic compounds of *V. opulus* fruit and fruit juice [25]. However, little information is available on these secondary metabolites in the bark and flower of this plant. In a previous study, phenolic composition determined by different spectrophotometric methods has shown that bark was characterized by the highest level of total phenolics, flavonoids, and proanthocyanidins as compared to flower and fruit [28]. Additionally, only eight and nine phenolic compounds were identified in *V. opulus* bark and flower, respectively. However, in the published article, the identification of individual phenolic compounds was based on the comparison of the retention time and UV-Vis absorption spectra of the peaks with the data for the standard substances. In the next study, the use of the UPLC-QTOF-MS technique allowed us to identify sixteen phenolic compounds in the crude extract of *V. opulus* bark [30]. In the present work, using the same UPLC-MS system, 26 and 17 phenolic compounds were determined in the flower and bark phenolic extracts, respectively (Figure 1). The extracts analyzed in the present study were obtained after purification of the crude extracts with the SPE (Solid Phase Extraction) method using a Sep-Pak C18 cartridge. The use of the SPE method was aimed at increasing the concentration of phenolic compounds in the extract as a result of removing non-phenolic compounds (e.g., proteins, organic acids) [22]. Next, the components of the obtained phenolic extracts were separated by liquid-liquid extraction between ethyl acetate and water. Ethyl acetate was selected for the fractionation procedure because previous work showed that the ethyl acetate fraction had a higher concentration of phenolic compounds and inhibited the activity of amylase and glucosidase more effectively compared to the hexane or methanol fraction [32,33,34].

Qualitative and quantitative analysis showed differences in phenolic compound compositions between extracts and fractions—Table 1 and Table 2. The decreasing rank of total phenolics for both bark and flower samples was as follows: acetate fraction > phenolic extract > water fraction. According to UPLC analysis, the bark samples showed the presence of three groups of phenolic compounds, such as flavanols, flavalignans, and hydroxycinnamic acids. For comparison, hydroxycinnamic acids, flavonols, and flavanon were found in flower samples, but flavanols and flavalignans were not. Quantitatively, flavanols dominated in all analyzed bark samples, while in flower samples, flavonols were the most prominent. The contribution of flavanols in the total content of phenolic compounds in bark samples ranged from 78.14 to 84.56% in the water fraction and extract, respectively (Table 1). However, flavonols constituted from 64.54 to 69.36% of the total phenolics in the acetate fraction and extract from flowers, respectively (Table 2). (+)-Catechin was identified as the main phenolic compound in the bark extract (64.90 mg/g of extract) and the bark acetate fraction (179.29 mg/g of fraction), whereas procyanidin B1 dominated in the bark water fraction (29.66 mg/g). Chlorogenic acid was the most prominent component in the flower extract (110.69 mg/g) and the flower water fraction (124.70 mg/g), while it was quantitatively the second compounds in the flower acetate fraction (178.47 mg/g). In this fraction, kaempferol 3-glucoside was present at the highest concentration (239.18 mg/g). There are only few literature data concerning the composition of the phenolic compounds of *V. opulus* bark [30,31]. Turek and Cisowski [31] have reported the presence of three cinnamic acid derivatives (caffeic, *p*-coumaric, and ferulic acids), four benzoic acid derivatives (gallic, protocatechuic, syringic, and 3,4,5-trimethoxybenzoic acids), two phenylacetic acid derivatives (3,4-dihydroxyphenylacetic and homogentisic acids), and two depsides (chlorogenic acid and ellagic acid). The current data are in line with our previous research, with the exception of the viburtinoside derivative that was only identified in *V. opulus* crude bark extract [30]. In addition, the previously described ethanolic crude bark extract contained less total flavanols (127.25 mg/g) and flavalignans (4.07 mg/g) than the phenolic bark extract (Table 1), but more total hydroxycinnamic acids (26.65 mg/g). The total phenolics in *V. opulus* bark crude extract depended on the type of extractant and ranged from 171.02 mg/g for water to 254.97 mg/g for 70% acetone [30]. Bubulica et al. [38] obtained much lower content, because the extract isolated from the bark with 80% methanol contained only 42.38 mg/g of total phenolics.

Even less information is available regarding the phenolic composition of *V. opulus* flower. Other studies confirm the presence of kaempferol derivatives and their significant contribution to the phenolic compounds identified in flowers [26]. It was also established that the content of total phenolics (Folin-Ciocalteu method) in *V. opulus* flower was 1.7 times lower than in fruit [29]. On the contrary, in the previous comparative studies we found comparable total phenolic content (35.1–39.8 mg/g DW) in flowers, bark, and fruits of *V. opulus* using the mentioned colorimetric method [28].

The total content of proanthocyanidins in the extracts and fractions was also determined by the spectrophotometric method after their acid depolymerization to the corresponding colored anthocyanidins (Table 3). The UPLC-MS method used in this study allowed only for the determination of proanthocyanidins with a low degree of polymerization (up to tetramer). The bark phenolic extract contained almost three times more proanthocyanidins than the flower phenolic extract (25.33 mg/g). Our previous research has shown that the content of total proanthocyanidins in *V. opulus* flower and bark was 2.2 and 10.3 mg/g DW, respectively [28]. Moreover, as a result of fractionation of the aqueous solution of extracts with ethyl acetate, the water fractions were richer in total proanthocyanidins than the acetate fractions and extracts. According to Saucier et al. [43], the proanthocyanidin oligomers are soluble in ethyl acetate while the polymers remain in the aqueous phase. Proanthocyanidins from grape seed and skin, acacia bark, persimmon peel and leaf, apple, and almond peel skin have been previously reported to inhibit α-amylase [44]. Additionally, proanthocyanidins with a high degree of polymerization compared to those with low degree had a greater effect on α-amylase activity in mice [45].

### 2.2. In Vitro Hydrolysis of Potato Starch in Presence of V. opulus Bark and Flower Extracts

The inhibition of the carbohydrates digestion, especially after intake of starchy-food, may suppress postprandial hyperglycemia and could be useful for treating diabetic patients. After carbohydrate intake, starch is first decomposed by α-amylases (salivary and pancreatic) to α-limit dextrins, maltotriose, and maltose, which are then broken down into glucose by a membrane-bound α-glucosidase [14,15]. Only the monosaccharides can enter into the blood circulation system and be utilized by human body. Therefore, one of the effective strategies to control postprandial hyperglycemia is delaying glucose absorption using α-amylase and α-glucosidase inhibitors. Among pharmaceuticals therapies currently available for the treatment of type 2 diabetes, acarbose, voglibose, and miglitol inhibit α-glucosidase in the lumen of the small intestine and retard the digestion of dietary carbohydrates to maintain postprandial blood glucose at normal levels [46].

**Figure 2:**
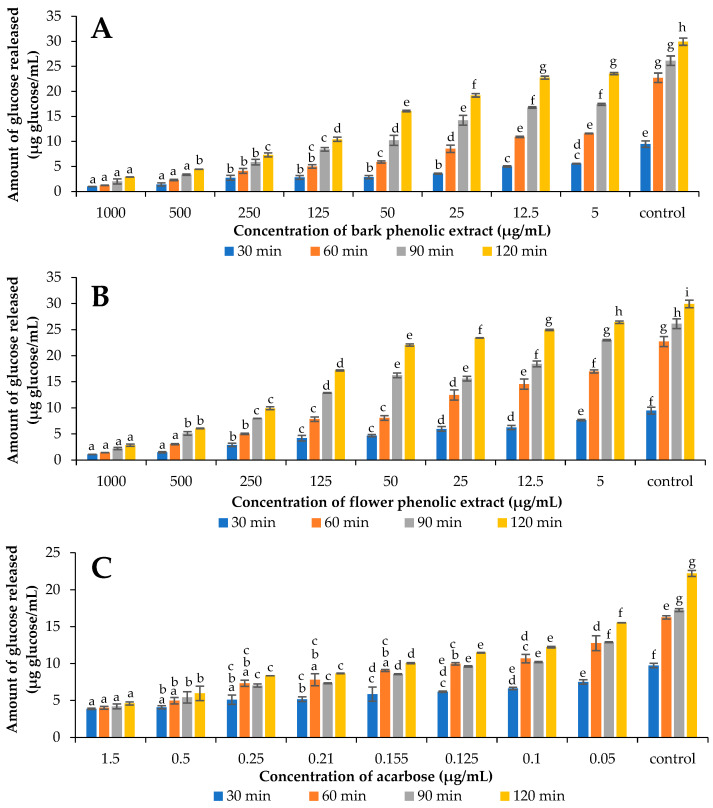
Amount of glucose released after 30, 60, 90, and 120 min of simulated intestinal digestion of potato starch without the presence of an inhibitor (control) and in the presence of various concentrations of bark (**A**) and flower (**B**) phenolic extracts or acarbose (**C**). The figure shows mean values ± standard deviations (*n* = 3). The means within 30, 60, 90, or 120 min with different letters differ statistically at *p* < 0.05.

**Figure 3:**
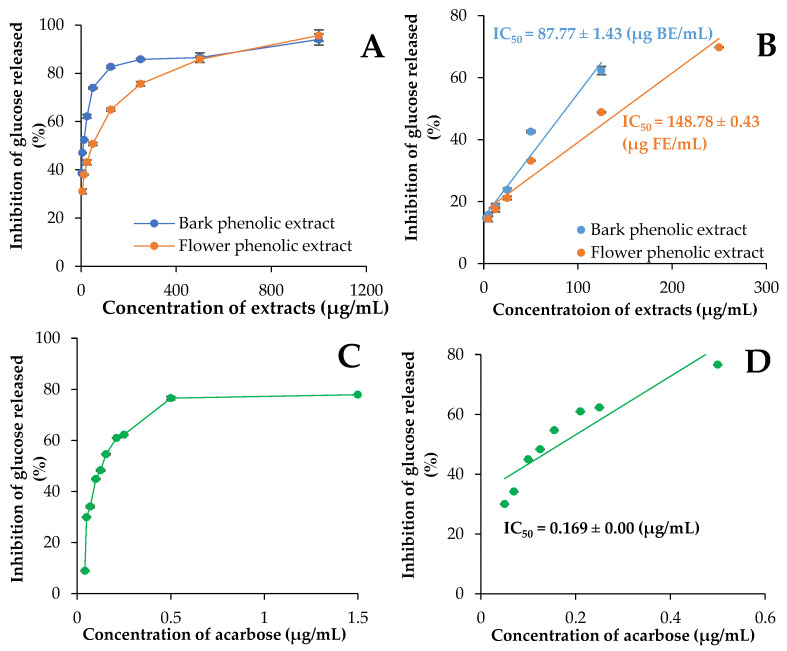
Inhibition of glucose released after 120 min simulated potato starch digestion in the presence of bark and flower phenolic extracts (**A**,**B**) and acarbose (**C**,**D**). The figure shows mean values ± standard deviations (*n* = 3).

In the present study, the influence of phenolic extracts from the bark and flower of *V. opulus* on the course of potato starch hydrolysis was investigated. For this purpose, a three-stage static model of simulated digestion was used. Potato starch was chosen as the substrate for α-amylase, as the same starch was also used in further experiments in which the amylase activity was determined. In Poland, potatoes are an important source of starch, and their consumption in 2019 was 99.42 kg/person/year. The consumption of cereals was comparable (107.65 kg/person/year), but lower for rice (about 80 kg/person/year) [47]. Moreover, the results of previous studies for *V. opulus* fruit extracts using potato or rice starch showed only differentiation in IC_50_ values, but the ranking of the samples with respect to inhibitory activity did not depend on the type of starch [22]. The progress of potato starch hydrolysis in the presence of *V. opulus* phenolic extracts and acarbose are shown in Figure 2. The rate of starch degradation was monitored by determining the amount of glucose released after 30, 60, 90, and 120 min of intestinal digestion. The increasing amount of glucose during the process confirmed the degradation of starch under the influence of α-amylase and α-glucosidase present in the system. Extending the time of simulated intestinal digestion from 30 to 120 min resulted in a more than three times increase in glucose concentration in control sample (without inhibitor). The addition of phenolic extracts from various parts of *V. opulus* (Figure 2A,B) as well as acarbose (Figure 2C) reduced the amount of glucose released. The effectiveness of the extracts and synthetic inhibitor increased with their concentration and digestion time. Moreover, the use of bark extract and flower extract in a dose greater than or equal to 125 and 500 µg/mL, respectively, reduced the amount of released glucose by more than 80%—Figure 3A. For comparison, acarbose showed a similar level of inhibition of potato starch hydrolysis at a concentration of 0.5 µg/mL (Figure 3C). The bark phenolic extract had a lower IC_50_ value (87.77 µg/mL) than flower phenolic extract (148.87 µg/mL)—Figure 3B. However, the concentration of extracts required to inhibit 50% of the potato starch hydrolysis was 500–900 times higher than the amount of acarbose with IC_50_ equal to 0.17 µg/mL (Figure 3D).

The digestion model used in this study does not fully reflect the actual conditions, for example due to the use of potato starch as a substrate instead of more complicated food matrix such as potatoes. In the case of digestion of food products containing starch, the course of its hydrolysis depends on, amongst other things, the type of starch, the presence of other nutrients and non-nutrients, the ingredients, as well as the technological processes used [48]. For example, in the presence of *Clitoria ternatea* L. flower extract, the highest degree of α-amylase inhibition was found in a system containing the potato flour, followed by glutinous rice, rice, wheat, corn, and cassava [10]. Nevertheless, the studies conducted in a more complex food matrix have indicated the influence of phenolic compounds on the starch digestion. For example, the addition of *Vaccinium bracteatum* leaf phenolic extract to rice extrudates significantly decreased *in vitro* starch digestibility [49]. Similarly, starch hydrolysis of cooked pasta enriched with black mulberry extract as well as wheat bread with *Clitoria ternatea* flower extract were significantly decreased [10,50].

### 2.3. Effects of V. opulus Phenolic Extracts from Bark and Flower on α-Amylase and α-Glucosidase Activity

**Figure 4:**
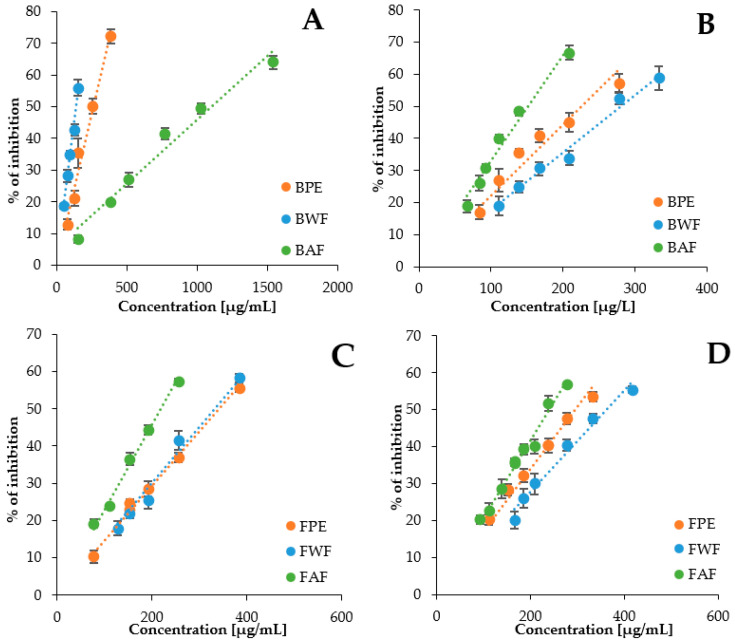
Inhibitory effects of *V. opulus* bark samples (**A**,**B**) and flower samples (**C**,**D**) on α-amylase activity in the presence of potato starch (**A**,**C**) and on α-glucosidase activity in the presence of maltose (**B**,**D**). BPE—bark phenolic extract, FPE—flower phenolic extract, BWF—bark water fraction, FWF—flower water fraction, BAF—bark ethyl acetate fraction, FAF—flower ethyl acetate fraction.

In order to explain the mechanism of the influence of *V. opulus* bark and flower components on delaying *in vitro* starch digestion, the effect of the analyzed extracts and their fractions on the activity of pancreatic α-amylase and α-glucosidase from rat intestinal was checked in the simple model systems. The measuring systems contained only the substrate, enzyme, and *V. opulus* sample, as well as a buffer conditioning the appropriate pH of the reaction. The α-amylase assay was performed using potato starch as substrate, while α-glucosidase activity was carried out using maltose as substrate. Pancreatic α-amylase is an endoglucosidase presents in pancreatic juice secreted into the intestinal lumen [51], while α-glucosidase is secreted from intestine epithelial cells [52]. The inhibitory effects of *V. opulus* samples from bark and flower were analyzed by the dose-effect plots (Figure 4A–D). Inhibitory activity of phenolic extracts and fractions, separated from extracts by liquid-liquid extraction with ethyl acetate, had a direct linear relationship between concentration and percentage inhibitory activity.

The extracts and fractions differed significantly (*p* < 0.05) in their IC_50_ values (Table 4). All bark and flower samples showed lower inhibitory activity against both enzymes compared to acarbose, with greater differences occurring with α-glucosidase. Moreover, the bark extract and its water fraction exceeded the corresponding flower samples in terms of inhibition of the activity of both analyzed enzymes. However, the comparison of the IC_50_ values of bark and flower acetate fractions showed that the acetate fraction of bark was a better α-glucosidase inhibitor while acetate flower fractions demonstrated higher activity against α-amylase. For comparison, the IC_50_ values of phenolic extract from *V. opulus* fruits were 61.51 µg/mL and 180.09 µg/mL against α-amylase and α-glucosidase, respectively [22]. This proves a higher inhibitory activity of the fruit components than the bark or flowers.

Based on the data from Table 1, Table 2 and Table 4, we cannot conclude about a possible correlation of the total content of phenolic compounds and the inhibitory activity in relation to the studied digestive enzymes. The less active flower samples were characterized by a 1.7 to 4.0 times higher content of phenolic compounds. On the other hand, the bark samples contained three times more proanthocyanidins (Table 3). Similarly, α-amylase and α-glucosidase inhibitory activities of *Canarium tramdenum* bark extracts showed no correlation with total phenolics [8]. The authors hypothesized that the observed activity may be the result of a synergistic interaction between the phenolics and terpenoids, with the composition of the phenolic compounds possibly being more important. According to Seeram [53], the biological properties of the phenolic compounds present in berry fruits could be correlated to the type of individual phenolics rather than the total phenolic content. The inhibitory activity of phenolic acids is enhanced with increasing the number of phenolic sub-structures. For example, the inhibitory effect of caffeic acid was enhanced 5-fold by combining with quinic acid to form chlorogenic acids [54].

Among all six samples tested, bark water fraction was the strongest inhibitor of porcine pancreatic α-amylase, while bark acetate fraction against α-glucosidase was indicated by the lowest IC_50_ values. Proanthocyanidins, especially procyanidin B1 and procyanidin trimer, were quantitatively the major components of the bark water fraction (Table 1 and Table 3). On the other hand, (+)-catechin was definitely dominant in the bark acetate fraction. Our previous study with *V. opulus* fruit extracts and fractions showed a significant contribution of chlorogenic acid, proanthocyanidin oligomers, and flavalignans in their anti-glucosidase activity, and proanthocyanidin polymers and dicaffeoylquinic acids in the inhibition of α-amylase activity [22]. For comparison, among water, methanol, and ethyl acetate extract from the stem bark of *Bridelia ferruginea,* only the last was found to inhibit α-glucosidase and was the strongest inhibitor of α-amylase [6]. This extract contained mainly flavan-3-ol monomers, ellagic acid, and its derivatives. The observed differences in the inhibitory activity of the extracts against digestive enzymes may result from the different affinities of polyphenolic compounds for amylase and glucosidase. For example, procyanidin dimer was a better α-amylase inhibitor than catechin, and the order was reversed in the α-glucosidase-containing system [55]. Moreover, the authors showed a higher efficiency of inhibiting the activity of these enzymes for rutin and quercetin than for the above-mentioned flavanols. Wang et al. [56] demonstrated the possibility of a synergistic interaction between flavonols in the inhibitory activity against α-glucosidase, but not against α-amylase.

### 2.4. Effects of V. opulus Bark and Flower Phenolic Extracts on α-Amylase and α-Glucosidase Spectra

**Figure 5:**
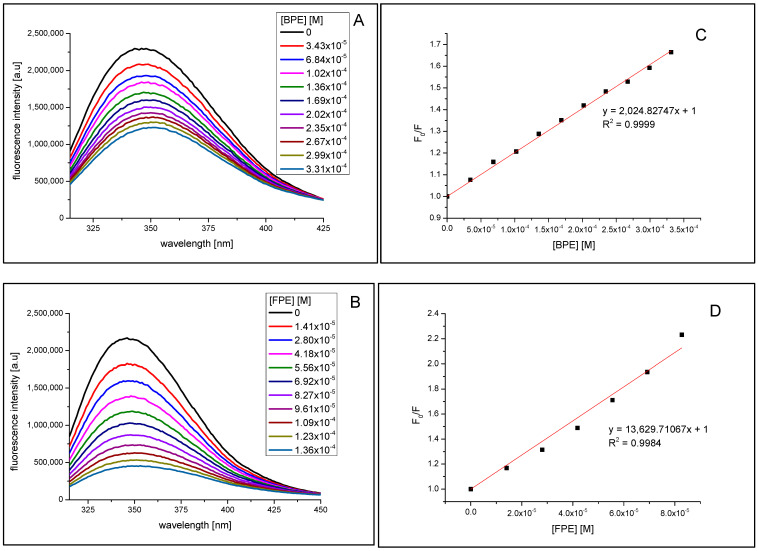
Fluorescence quenching spectra of α-amylase by bark phenolic extract (BPE) (**A**) and flower phenolic extract (FPE) (**B**), λ_ex_ = 295 nm, the BE concentration was expressed in (+)-catechin equivalent, the FE concentration was expressed in chlorogenic acid equivalent. The Stern-Volmer plots for α-amylase fluorescence quenching by BPE (**C**) and by FPE (**D**).

The fluorescence spectra of α-amylase and α-glucosidase at different concentrations of bark phenolic extract and flower phenolic extract are shown in Figure 5. It was evident that with the increasing concentration of both extracts, the fluorescence intensity of both enzymes reduced progressively, which is indicative of molecular interactions between extract components and enzyme.

The docking study demonstrated that phenolic compounds identified in *V. opulus* bark and flower extracts such as chlorogenic acid, neochlorogenic acid, cryptochlorogenic acid, coumaroylquinic acid, caffeic acid, catechin, epicatechin, and rutin had a binding affinity with α-amylase and α-glucosidase [32,33,57,58]. Moreover, neochlorogenic acid demonstrated strong α-glucosidase catalytic efficiency in terms of binding affinity and hydrogen bonding interaction.

**Figure 6:**
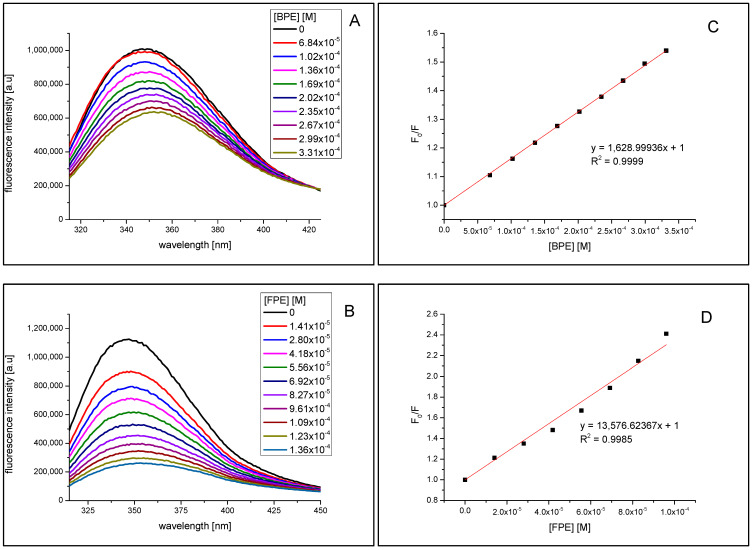
Fluorescence quenching spectra of α-glucosidase by bark phenolic extract (BPE) (**A**) and by flower phenolic extract (FPE) (**B**), λ_ex_ = 295 nm. The Stern-Volmer plots for α-glucosidase fluorescence quenching by BPE (**C**) and by FPE (**D**).

From Figure 5A,B as well as Figure 6A,B it can be seen that addition of the bark phenolic extract (BPE) and flower phenolic extract (FPE) to α-amylase and α-glucosidase solutions resulted in tryptophan fluorescence quenching. Moreover, a slight red shift of the tested enzymes emission spectra maximum may be observed upon increasing concentration of the quencher. Due to the presence of this alteration to the fluorescence maximum, the Stern-Volmer plots (Figure 5C,D and Figure 6C,D) were obtained from the integrated fluorescence intensities (the area under the spectrum with the wavelength range of 310–450 nm). The determined value of the Stern–Volmer constant for α-amylase quenching by bark phenolic extract and flower phenolic extract was 2.025 ± 0.002 × 10^3^/M and 1.363 ± 0.049 × 10^4^/M, respectively. The value of the Stern–Volmer constant for α-glucosidase quenching by bark and flower phenolic extracts was 1.629 ± 0.006 × 10^3^/M and 1.358 ± 0.041 × 10^4^/M, respectively. The higher values of the Stern–Volmer constant for both enzymes quenching by flower phenolic extract as compared to that for bark phenolic extract indicates that the components of *V. opulus* flower more effectively bind to α-amylase and α-glucosidase although. Results presented in Section 2.3 showed that the bark extract inhibited the hydrolytic activity of these enzymes to a greater extent. This may indicate a complex mechanism of the influence of the components of the polyphenol extract from the bark and flowers on the activity of amylase and glucosidase and, consequently, also the course of starch hydrolysis.

## 3. Materials and Methods

### 3.1. Standards and Reagents

Intestinal acetone powder from a rat source of α-glucosidase (EC 3.2.1.20), α-amylase from porcine pancreas type VI-B (EC 3.2.1.1), pancreatin from porcine pancreas, pepsin from the gastric mucosa of pigs, bile from bovine and ovine, TRIS-HCl, acarbose, caffeic acid, chlorogenic acid, (+)-catechin, cinchonine, (+)-catechin, (−)-epicatechin, kaempferol 3-glucoside, naringin, sodium chloride, maltose, formic acid, methanol, and acetonitrile were obtained from Sigma Aldrich (Steinheim, Germany). Acetone, ethanol, ethyl acetate, hydrochloric acid, sodium hydroxide, sodium bicarbonate, iodine, potassium iodide, disodium phosphate, and monosodium phosphate were purchased from Chempur (Piekary Śląskie, Poland). Potato starch and calcium chloride were purchased from POCH (Gliwice, Poland) and glucose test from Biomaxima SA (Lublin, Poland). Quercetin 3-glucoside, quercetin 3-rutinoside, quercetin 3-rhamnoside, and isorhamnetin 3-glucoside were obtained from Extrasynthese (Lyon, France). Procyanidin C1, procyanidin B1, procyanidin B2, neochlorogenic acid, cryptochlorogenic acid, and 3,5-dicaffeoylquinic acid were purchased from PhytoLab (Vestenbergsgreuth, Germany). Kaempferol was purchased from ICN Biomedicals (Costa Mesa, CA, USA). Ultrapurity water was prepared in the laboratory using a Simplicity Water Purification System (Millipore, Marlborough, MA, USA).

### 3.2. Plant Material and Phenolic Extracts Preparation

**Figure 7:**
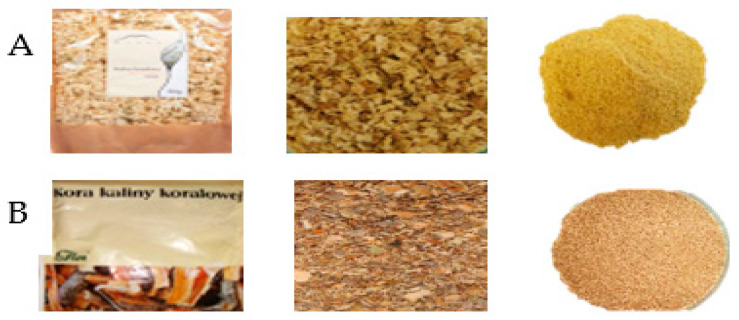
Plant materials used to obtain the phenolic-rich extract of *V. opulus* flower (**A**) and bark (**B**). From the left: a unit package of a commercial product, dried material, and ground dried material.

Commercial samples of the dried flowers and bark of *V. opulus* were bought from a Polish providers “Nanga Przemysław Figura” (Złotów, Poland) and “Flos” (Makrsko, Poland), respectively. Prior to the extraction, the plant material was grounded in a coffee grinder (Figure 7). Flowers (50 g) were extracted with 70% acetone (1:20, *w*/*v*), and bark (50 g) with 70% ethanol (1:20, *w*/*v*), on a magnetic stirrer at room temperature for 3 h. Then, the mixtures were incubated at room temperature for 18 h, followed by the extraction on a magnetic stirrer at room temperature for 3 h. After centrifugation at 5000 rpm for 10 min, the supernatants were evaporated at 40 °C under reduced pressure in order to remove organic solvent, and lyophilized to obtain the crude extracts. For phenolics-rich extracts, 250 mg of the crude extract in 5 mL of water was loaded onto a Sep-Pak C18 cartridge (10 g capacity, Waters Corp., Milford, MA, USA) that was previously activated with methanol (60 mL) and water (60 mL). The column was washed with water in order to eliminate carbohydrates, proteins, and other polar compounds. The phenolic compounds were eluted with methanol (60 mL), which was evaporated under reduced pressure (T < 40 °C). After dissolving in water, the dry residue was freeze-dried to afford phenolic extract from flower (FPE) or from bark (BPE). The SPE (solid phase extraction) purification procedure was repeated five times for each crude extract. The obtained dry extracts were stored at 4 °C until use. The extraction yields calculated as the ratio between the total mass of phenolic-rich extract and the mass of dried plant material used for extraction were 19.30% and 11.56% for FPE and BPE, respectively.

Subsequently, a portion of each extract (300 mg) was suspended in 30 mL of water, and partitioned with ethyl acetate (30 mL × 3). The organic phases were evaporated to dryness, solubilized with water, and lyophilized. The obtained samples were named bark ethyl acetate fraction (BAF) and flower ethyl acetate fraction (FAF). The water phases were also concentrated and lyophilized to afford bark water fraction (BWF) and flower water fraction (FWF).

### 3.3. Identification and Content of Individual Phenolic Compounds

UPLC-MS analysis was performed on an ultra-performance liquid chromatograph (Waters Acquity UPLC system, Milford, MA, USA) equipped with a binary pump, an autosampler, a column compartment, and a diode array detector. Briefly, samples were eluted with a gradient of solvent A (4.5% formic acid in ultrapure water) and B (acetonitrile) on an Acquity UPLC HSS T3 C18 column (150 × 2.1 mm, 1.8 μm; Waters) operating at 30 °C, as described in the previous work [22]. The gradient program was as follows: initial conditions 99% (A), 12 min 75% (A), 12.5 min 100% (B), 15.0 min 99% (A). The flow rate was 0.45 mL/min and the injection volume was 5 μL. The identification of phenolic compounds by UPLC-QTOF-MS method was described in detail previously [30]. The mass spectrometer was operating in the negative mode for a mass range of 150–1500 Da, fixed source temperature at 100 °C, desolvation temperature 250 °C, desolvation gas flow of 600 L/h, cone voltage of 45 V, capillary voltage of 2.0 kV, collision energy 50 V. Leucine enkephalin was used as a lock mass. The instrument was controlled by Mass-Lynx^TM^ V 4.1 software. Procyanidin B1, B2, and C1, (+)-catechin, (−)-epicatechin, caffeic acid, neochlorogenic acid, chlorogenic acid, cryptochlorogenic acid, 3,5-dicaffeoylquinic acid, quercetin 3-rutinoside, quercetin 3-glucoside, kaempferol, kaempferol 3-glucoside, and isorhamnetin 3-glucoside were confirmed by comparison with authentic standards. Other compounds were tentatively identified on the basis of their UV-Vis spectra, MS, and MS^2^ properties in comparison with the literature data.

The content of neochlorogenic acid, chlorogenic acid, cryptochlorogenic acid, caffeic acid, 3,5-dicaffeoylquinic acid, (+)-catechin, (−)-epicatechin, procyanidin B1, procyanidin B2, procyanidin C1, quercetin 3-glucoside, quercetin 3-rutinoside, quercetin 3-rhamnoside, isorhamnetin 3-glucoside, kaempferol 3-glucoside, and kaempferol were quantified using corresponding standard calibration curves. A quantitative analysis of other phenolics was based on the standards as follow: chlorogenic acid was used for the hydroxycinnamic acid derivatives, (+)-catechin for (epi)-catechin hexoside, procyanidin C1 for procyanidin trimer and tetramer, procyanidin B1 for procyanidin dimer, cinchonine for cinchonain Ix and cinchonain IIx derivatives, naringin for eriodictyol hexoside, quercetin 3-glucoside for quercetin derivatives, isorhamnetin 3-glucoside for isorhamnetin derivatives, and kaempferol 3-glucoside for kaempferol derivatives. The results were expressed as mg per gram of extract or fraction.

### 3.4. Total Proanthocyanidins Content

The content of total proanthocyanidins was determined after their acid depolymerization to the corresponding anthocyanidins as described by Rösch et al. [59] and calculated by the molar extinction coefficient of cyanidin (ε = 17,360 L/mol × cm and molar mass 287 g/mol), and was expressed as mg of cyanidin equivalents (CYE)/g of *V. opulus* samples.

### 3.5. Simulated In Vitro Digestion of Potato Starch

The simulated potato starch digestion was modified based on the methodology described by Bellesia et al. [60] and Yang et al. [61]. The *in vitro* digestion process consisted of three stages: oral, gastric, and intestinal digestion, the course of which is described in Table 5. For the simulated digestion process, the following solutions were prepared: potato starch solution (0.5 g was gelatinized in 20 mL of water for 2.5 min from the moment of boiling. After cooling, the volume of the solution was made up to 20 mL with water.), and α-glucosidase solution (31.25 mg acetone intestinal powder from rat with 1.2 mL of 0.9% NaCl solution extracted in an ultrasonic cleaner for 30 s in an ice bath, then 30 s without ultrasonic. This step was repeated 12 times. The mixture was centrifuged in 4 °C, 3000 rpm in 30 min and the supernatant was made up to a volume of 25 mL).

All digestion steps were carried out in a water shaking bath. To determine the amount of glucose released from starch, after 30, 60, 90, and 120 min of simulated intestinal digestion, 1 mL of digestion was taken into a tube containing 0.4 mL of a commercial test. The samples were incubated at 37 °C for 10 min, after which the absorbance was measured at a wavelength of 500 nm using spectrophotometer SP-830 Plus (Metertech, Taipei City, Taiwan). Glucose content was determined on the basis of the regression curve. Control sample containing water instead of the *V. opulus* phenolic extracts, and blanks containing water instead of enzymes solution, were also prepared. Simulated digestion of potato starch was also performed with different concentration of acarbose. The IC_50_ values (concentration of the extract that caused 50% inhibition of starch hydrolysis) were calculated from a regression curve of the percentage (%) inhibition of glucose released against various concentration of the extract.

### 3.6. α-Amylase Inhibition Assay

The α-amylase inhibition assay was based on a previously-described spectrophotometric method [22]. All reagents was prepared in 0.1 M phosphate buffer containing 6 mM CaCl_2_ (pH 6.9). Briefly, 20 µL of diluted *V. opulus* samples and 40 µL of gelatinized potato starch (0.83 g/L) solution were mixed with 20 µL of α-amylase (0.1 mg/mL) in a 96-well plate. After incubation at 37 °C for 10 min, the reaction was stopped by addition of 80 mL of 0.4 M HCl, followed by 100 µL of 5 mM I_2_ in 5 mM KI. The absorbance was read at 600 nm using a microplate reader (Synergy2, BioTek Instruments Inc., Winooski, VT, USA). Acarbose was used as positive control. Each sample was measured in triplicate. The IC_50_ values (concentration of the extract or fraction that caused 50% inhibition) were calculated from a regression curve of the percentage (%) inhibitions against various concentrations of the samples.

### 3.7. α-Glucosidase Inhibition Assay

The assessment of the α-glucosidase inhibitory activity was according to our previous work [22]. Briefly, 125 mg of rat intestinal acetone powder was mixed with 2.5 mL of 0.9% NaCl solution and enzyme isolation was performed in an ultrasonic bath as described in Section 3.5. 50 μL of enzyme supernatant (diluted twice) was mixed with 50 μL of diluted *V. opulus* samples. After incubation at 37 °C for 10 min, 50 μL of maltose (0.1 M in 0.1 M phosphate buffer pH 6.9) was added and incubated in a 96-well microplate at 37 °C for 20 min. The reaction was stopped by adding 150 µL of 2 M Tris–HCl buffer (pH 7.0). The concentrations of glucose released from the reaction mixtures were determined by the commercial glucose test. Acarbose was used as a positive control. The IC_50_ values were calculated as described in Section 3.6. by regression analysis.

### 3.8. Fluorescence Measurements

The effect of *V. opulus* bark and flower phenolic-rich extracts on fluorescence spectra of α-amylase and α-glucosidase at different concentrations of extracts (from 0.014 to 0.136 mM) were performed using FluoroMax 4 (Jobin Yvon Spex) spectrofluorometer (Horiba Scientific, Piscataway, NJ, USA) according to the method described previously [22]. In brief, 2.5 mL solution containing 2 × 10^−6^ M α-amylase and 1.3 × 10^−6^ M α-glucosidase in 0.01 M PBS (pH 7.4), was titrated by successive additions of extracts to give a final concentration 1.36 × 10^−4^ M for flower phenolic extract and to 3.31 × 10^−4^ M for bark phenolic extract. The concentrations of bark and flower phenolic extracts were expressed as (+)-catechin (M = 290.26 g/mol) and chlorogenic acid (M = 354.31 g/mol) equivalents, respectively. The fluorescence spectra of enzymes and their changes upon adding increasing amounts of extracts were recorded in the wavelength range of 315–450 nm upon excitation at 295 nm.

### 3.9. Statistical Analysis

All samples were assayed in triplicate and results are given as the mean ± standard deviation using Microsoft Excel XP. Significance differences were calculated using one-way analysis of variance (ANOVA) using Statistica Ver. 6.0 (TIBCO Software Inc., Palo Alto, CA, USA). Difference among means was determined by Tukey’s test at a significance level of *p* < 0.05.

## 4. Conclusions

*V. opulus* bark and flower phytochemicals were first assessed in terms of their antidiabetic potential, evaluated as inhibitory activity against α-amylase and α-glucosidase as well as potato starch hydrolysis in static simulated digestion model. It was shown that phenolic extract from the bark of *V. opulus* was superior to phenolic extract from flowers in terms of inhibiting the activity of the analyzed carbohydrate digestive enzymes and enzymatic decomposition of starch. This may be related to the presence of unidentified flavanols and flavalignans in the flower extract and, above all, a higher content of proanthocyanidins. In addition, it was observed that α-amylase inhibitors from bark showed a higher affinity for water, and α-glucosidase inhibitors for ethyl acetate. Of course, the activity of the analyzed extracts and fractions, which are a mixture of structurally diverse phenolic compounds, is the resultant of the activity of individual components, probably also of non-phenolic phytochemicals. The interactions between the components of the analyzed samples should also be taken into account. Our results suggest that the bark of *V. opulus* is a more valuable source of carbohydrate digestive enzyme inhibitors and could be used as an ingredient in nutraceuticals and functional foods for diabetics. Nevertheless, further studies in more complex systems and a commercially viable purification procedure are required if *V. opulus* bark phytochemicals are going to find widespread practical application.

## Figures and Tables

**Table 1:** The phenolic compounds content in *V. opulus* bark samples.

Peak	R_t_ (min)	λ_max_ (nm)	[M − H]^−^ (*m*/*z*)	MS/MS (*m*/*z*)	Phenolic Compound	Extract	Acetate Fraction	Water Fraction
						mg/g of Extract or Fraction		
Flavanols								
1	4.61	281	577	125,161,255	Procyanidin B1	36.56 ± 0.02 ^c^	22.16 ± 0.06 ^a^	29.66 ± 1.18 ^b^
2	5.11	281	865	407,289,125	Procyanidin trimer I ^a,1^	28.50 ± 0.02 ^c^	22.86 ± 0.12 ^b^	14.22 ± 0.02 ^a^
3	5.26	278	289	109,159,173	(+)-Catechin	64.90 ± 0.03 ^b^	179.29 ± 0.14 ^c^	6.70 ± 0.16 ^a^
4	5.45	279	865	407,289,125	Procyanidin trimer II ^a,1^	12.75 ± 0.07 ^c^	10.37 ± 0.02 ^b^	6.12 ± 0.20 ^a^
5	6.12	279	577	125,161,255	Procyanidin B2	10.58 ± 0.06 ^c^	8.09 ± 0.11 ^a^	9.50 ± 0.10 ^b^
6	6.7	279	1153	287,407,125	Procyanidin tetramer I ^a,1^	4.12 ± 0.02 ^b^	8.10 ± 0.03 ^c^	1.92 ± 0.09 ^a^
7	6.91	279	289	109,159,173	(−)-Epicatechin	7.66 ± 0.01 ^a^	18.20 ± 0.01 ^b^	-
8	7.29	279	865	407,243,289	Procyanidin C1	5.41 ± 0.09 ^b^	6.85 ± 0.16 ^c^	2.50 ± 0.27 ^a^
9	7.61	279	577	125,161,255	Procyanidin dimer ^b,1^	9.68 ± 0.05 ^a^	29.16 ± 0.05 ^b^	-
Total flavanols						180.16 ± 0.37 ^b^	305.08 ± 0.70 ^c^	70.62 ± 2.02 ^a^
Flavalignans								
10	7.39	279	739	177,289,161	Cinchonain IIx ^c,2^	4.28 ± 0.09 ^b^	8.63 ± 0.01 ^c^	1.10 ± 0.04 ^a^
11	9.23	281	451	176,191,269	Cinchonain Ix ^c,2^	2.28 ± 0.06 ^a^	3.10 ± 0.01 ^b^	-
12	10.88	281	451	189,161	Cinchonain Ix ^c,2^	1.24 ± 0.01 ^a^	6.78 ± 0.03 ^b^	-
Total flavalignans						7.80 ± 0.16 ^b^	18.51 ± 0.05 ^c^	1.10 ± 0.04 ^a^
Hydroxycinnamic Acids								
13	4.05	324	353	134,135, 191	Neochlorogenic acid	1.16 ± 0.00 ^b^	0.26 ± 0.00 ^a^	-
14	5.71	326	353	191,133	Chlorogenic acid	17.15 ± 0.00 ^a^	22.74 ± 0.01 ^c^	17.26 ± 0.01 ^b^
15	5.9	324	353	191,133	Cryptochlorogenic acid	1.07 ± 0.00 ^b^	0.59 ± 0.00 ^a^	1.18 ± 0.01 ^c^
16	10.79	325	515	191,135	3,5-Dicaffeoylquinic acid	2.06 ± 0.00 ^c^	0.45 ± 0.00 ^b^	0.19 ± 0.00 ^a^
17	11.63	325	515	191,135	Dicaffeoylquinic acid ^d,3^	3.56 ± 0.01 ^a^	19.95 ± 0.01 ^b^	-
Total hydroxycinnamic acids						25.00 ± 0.01 ^b^	43.99 ± 0.02 ^c^	18.63 ± 0.02 ^a^
TOTAL PHENOLICS						212.96 ± 0.54 ^b^	367.58 ± 0.77 ^c^	90.35 ± 2.08 ^a^

The content expressed as equivalents of: ^a^—procyanidin C1, ^b^—procyanidin B1, ^c^—cinchonine, ^d^—chlorogenic acid. Identification of phenolic compounds on the basis of: ^1^—[35]; ^2^—[36]; ^3^—[37]. The means within a same raw with different letters differ statistically at *p* < 0.05.

**Table 2:** The phenolic compounds content in *V. opulus* flower samples.

Peak	R_t_ (min)	λ_max_ (nm)	[M − H]^−^ (*m*/*z*)	MS/MS (*m*/*z*)	Phenolic Compound	Extract	Acetate Fraction	Water Fraction
						mg/g of Extract or Fraction		
Flavanon								
1	9.37	281	449	135,151	Eriodictyol hexoside ^a,1^	0.59 ± 0.00 ^a^	0.71 ± 0.00 ^b^	-
Hydroxycinnamic Acids								
2	5.67	326	353	191,133	Chlorogenic acid	110.69 ± 0.15 ^a^	178.47 ± 0.08 ^c^	124.70 ± 0.06 ^b^
3	5.89	326	353	191,133	Cryptochlorogenic acid	1.29 ± 0.00 ^b^	1.33 ± 0.02 ^c^	0.90 ± 0.00 ^a^
4	6.07	324	179	108,134,191	Caffeic acid	1.03 ± 0.00 ^a^	1.13 ± 0.00 ^b^	-
5	7.38	326	335	135	Caffeoylshikimic acid ^b,2^	4.89 ± 0.18 ^a^	28.22 ± 0.01 ^b^	-
6	8.29	305	337	191,117,127	*p*-Coumaroylquinic acid ^b,1^	1.48 ± 0.11 ^a^	2.80 ± 0.00 ^b^	-
7	10.75	327	515	191,135	3,5-Dicaffeoylquinic acid	4.44 ± 0.09 ^a^	5.60 ± 0.00 ^b^	-
Total hydroxycinnamic acids						123.82 ± 0.53 ^a^	217.55 ± 0.11 ^c^	125.60 ± 0.06 ^b^
Flavonols								
8	6.34	352	625	299,271	Quercetin dihexoside ^c,3^	6.48 ± 0.01 ^a^	-	10.27 ± 0.01 ^b^
9	6.64	338	771	285	Quercetin dihexoside ^c,4^	1.05 ± 0.03 ^a^	-	1.87 ± 0.00 ^b^
10	7.15	322	609	283,255	Kaempferol 3-sophoroside ^d,3^	69.12 ± 0.40 ^b^	7.64 ± 0.00 ^a^	90.67 ± 1.05 ^c^
11	7.32	345	755	285	Quercetin dihexoside ^c,4^	6.41 ± 0.01 ^a^	-	8.77 ± 0.03 ^b^
12	7.58	352	639	331,300,270	Laricitin 3-rutinoside ^c,3^	4.06 ± 0.00 ^b^	0.32 ± 0.00 ^a^	6.00 ± 0.00 ^c^
13	8.08	343	651	283,255	Quercetin 3-(acetyl)-rutinoside ^c,4^	1.71 ± 0.00 ^a^	-	1.73 ± 0.00 ^b^
14	8.19	338	593	283,255,161	Kaempferol hexoside ^d,3^	0.78 ± 0.05 ^a^	-	1.10 ± 0.00 ^b^
15	9.11	352	595	271,255,300	Quercetin 3-sambubioside ^c,5^	1.01 ± 0.00 ^a^	-	1.51 ± 0.00 ^b^
16	9.71	352	609	271,255,300	Quercetin 3-rutinoside	7.49 ± 0.01 ^b^	1.19 ± 0.01 ^a^	9.53 ± 0.05 ^c^
17	9.99	352	463	271,255,243	Quercetin 3-glucoside	17.62 ± 0.01 ^b^	33.67 ± 0.02 ^c^	8.10 ± 0.00 ^a^
18	10.23	347	579	255,227,285	Quercetin pentosyldeoxyhexoside ^c,6^	13.98 ± 0.01 ^b^	3.59 ± 0.09 ^a^	19.73 ± 0.00 ^c^
19	10.62	354	505	271,255,243	Quercetin 3-(acetyl)-galactoside ^c,4^	5.55 ± 0.01 ^b^	6.87 ± 0.00 ^c^	3.32 ± 0.01 ^a^
20	10.88	347	593	255,227,285	Kaempferol 3-rutinoside ^d,4^	13.34 ± 0.18 ^b^	9.45 ± 0.00 ^a^	15.31 ± 0.00 ^c^
21	11.16	360	447	227,255,183	Kaempferol 3-glucoside	81.46 ± 0.06 ^b^	239.18 ± 0.03 ^c^	42.21 ± 0.05 ^a^
22	11.59	352	477	243,271,199	Isorhamnetin 3-glucoside	11.65 ± 0.24 ^b^	23.97 ± 0.00 ^c^	3.98 ± 0.00 ^a^
23	12.05	348	489	227,255	Kaempferol 3-(acetyl)-glucoside ^d,4^	30.74 ± 0.14 ^b^	64.75 ± 0.04 ^c^	7.95 ± 0.00 ^a^
24	12.28	345	489	227,255	Kaempferol 3-(acetyl)-glucoside ^d,4^	1.28 ± 0.00 ^a^	2.81 ± 0.00 ^b^	-
25	12.53	354	519	243,271,285	Isorhamnetin 3-(acetyl)-glucoside ^e,3^	2.56 ± 0.01 ^b^	4.60 ± 0.00 ^c^	0.91 ± 0.00 ^a^
26	13.14	366	285	182,117,227	Kaempferol	5.29 ± 0.03	-	-
Total flavonols						281.58 ± 1.20 ^b^	398.04 ± 0.19 ^c^	232.97 ± 1.20 ^a^
Total phenolics						405.99 ± 1.73 ^b^	616.30 ± 0.30 ^c^	358.57 ± 1.26 ^a^

The content expressed as equivalents of: ^a^—naringin, ^b^—chlorogenic acid, ^c^—quercetin 3-glucoside, ^d^—kaempferol 3-glucoside, ^e^—isorhamnetin 3-glucoside. Identification of phenolic compounds on the basis of: ^1^—[37]; ^2^—[35]; ^3^—[39]; ^4^—[40]; ^5^—[41]; ^6^—[42]. The means within a same raw with different letters differ statistically at *p* < 0.05.

**Table 3:** Total proanthocyanidins content (mg/g) of the *V. opulus* bark and flower samples.

Scheme	Bark	Flower
Phenolic extract	71.85 ± 3.50 ^a^	25.33 ± 1.23 ^a^
Acetate fraction	73.04 ± 3.83 ^a^	23.27 ± 2.15 ^a^
Water fraction	127.29 ± 1.72 ^b^	42.53 ± 2.19 ^b^

The table shows mean values ± standard deviations (*n* = 3). The means in column within bark and flower samples with different letters differ statistically at *p* < 0.05.

**Table 4:** IC_50_ values (µg/mL) of the *V. opulus* bark and flower samples, and acarbose for the inhibition of α-amylase and α-glucosidase.

	Sample	α-Amylase	α-Glucosidase
Bark	Phenolic extract	260.75 ± 2.51 ^d^	217.03 ± 11.17 ^c^
	Acetate fraction	985.80 ± 19.00 ^f^	164.85 ± 2.75 ^b^
	Water fraction	140.86 ± 4.98 ^b^	267.05 ± 9.70 ^e^
Flower	Phenolic extract	351.87 ± 3.02 ^e^	300.29 ± 12.65 ^f^
	Acetate fraction	224.70 ± 3.78 ^c^	243.08 ± 3.90 ^d^
	Water fraction	337.15 ± 7.86 ^e^	346.14 ± 4.44 ^g^
Acarbose		13.33 ± 0.17 ^a^	0.051 ± 0.001 ^a^

The table shows mean values ± standard deviations (*n* = 3). The means in column within bark and flower samples and acarbose with different letters differ statistically at *p* < 0.05.

**Table 5:** Composition of the mixtures of the three-stage simulated *in vitro* digestion of potato starch.

**Oral Digestion; Incubation Conditions: 37 °C, 2 min**
0.05–20 mg of bark or flower phenolic-rich extract
1 mL of water
1 mL of gelatinized potato starch (25 g/L)
2.5 mL saliva solution (prepared according to [57])
0.5 mL α-amylase solution (0.1 mg/mL)
**Gastric Digestion; Incubation Conditions: 37 °C, 2 h**
4.5 mL gastric solution (2 g NaCl in 0.7% HCl in water, pH 1.2)
0.5 mL pepsin solution (3.2 mg/mL)
pH correction to a value of 2.0 with 2 M NaOH
**Intestinal Digestion; Incubation Conditions: 37 °C, 2 h**
5 mL of water
pH correction to a value of 6.0 with 2 M NaOH followed to 7.5 with 1 M NHCO_3_
The volume of the sample was adjusted to 16.4 mL with water
1 mL of bile salts (100 mg/mL)
2 mL of α-glucosidase solution
0.6 mL of pancreatin solution (0.04 mg/mL)

## Data Availability

Not applicable.

## Sample Availability

Samples of the compounds are not available from the authors.
